# Audit of Antipsychotic Monitoring in an Old Age Community Mental Health Team in Accordance With NICE Guidelines

**DOI:** 10.1192/bjo.2025.10699

**Published:** 2025-06-20

**Authors:** Serife Dilara Yozgatli, Felix Clay

**Affiliations:** CPFT, Cambridge, United Kingdom

## Abstract

**Aims:** Antipsychotic monitoring is crucial for identifying and managing side effects, improving treatment compliance, and reducing risks associated with long-term use. NICE guidelines recommend routine monitoring to enhance quality of life and prevent disengagement due to adverse effects. This audit assesses compliance with these guidelines within an Old Age Community Mental Health Team (CMHT). This was also discussed in MDT, as well as with patients and carers to have a better understanding of patient experience and how we can enhance antipsychotic monitoring.

**Methods:** We have registered our audit with Clinical Effectiveness Team at Cambridgeshire and Peterborough NHS Trust. We screened 101 patients under Ely Neighbourhood Team. We included patients with Schizophrenia, Schizoaffective Disorder, Bipolar Disorder, or Delusional Disorder currently on antipsychotic medication. Of the 18 patients identified with these diagnoses, 17 were on antipsychotics. We have screened their notes in the last 12 months for Body Mass Index (BMI), ECG, Complete Blood Count (CBC), Electrolytes (U&E),Blood Lipids, HbA1c, Pulse and Blood Pressure, Liver Function Tests (LFT), Emergence of Extrapyramidal Side Effects (EPSE) or Movement Disorders.

**Results:** 17 patients were included in the audit. Patients were between ages 66 and 84. Of them 5 were males and 12 females. Of the 17 patients 6 (35%) of them had Schizophrenia, 3 (18%) of them had Paranoid Schizophrenia, 3 (18%) of them had Delusional Disorder and 5 (29%) of them had Bipolar Disorder. Within the last 12 months, all patients on antipsychotics were offered monitoring; 1 patient declined. 94% had blood work monitoring. 100% had pulse and BP recorded. 29% (5 patients) did not have an ECG, despite being on medications requiring ECG monitoring. 11 patients (65%) were not asked about EPSE/movement disorders. Of the 6 patients asked about EPSE, 66% (4) were asked in outpatient reviews, and 33% (2) were asked as inpatients in psychiatric units.

**Conclusion:** Despite good compliance with most aspects of antipsychotic monitoring, ECG and movement disorder evaluations require improvement in elderly CMHT cohorts. We recommend psychiatrists to work collaboratively with GPs to enhance antipsychotic monitoring.

